# Photosynthesis and Carbon Metabolism in Higher Plants and Algae

**DOI:** 10.3390/plants14142161

**Published:** 2025-07-13

**Authors:** Natalia N. Rudenko

**Affiliations:** Institute of Basic Biological Problems, Federal Research Center “Pushchino Scientific Center for Biological Research of the Russian Academy of Sciences”, Pushchino 142290, Russia; nataliacherry413@gmail.com

## 1. Background

Photosynthesis is the most important process on Earth because it provides molecular oxygen and enables the growth of higher plants, algae, and cyanobacteria by allowing them to produce organic matter from carbon dioxide. Inorganic carbon is the substrate for a key reaction in the dark stage of photosynthesis, involving the carboxylation of ribulose-1,5-bisphosphate by the enzyme ribulose bisphosphate carboxylase/oxygenase (RuBisCO), which is the most abundant protein in plant cells.

For CO_2_ fixation, autotrophs follow three different photosynthetic strategies, namely, C3, C4, or CAM photosynthesis. Most plants, including algae, use the C3 type of photosynthesis. The RuBisCO enzyme plays a key role in the assimilation of atmospheric CO_2_, but it is an inefficient enzyme with a low turnover rate (for a review, see [1]). Under various stress conditions that lead to stomatal closure and insufficient CO_2_ supply, this enzyme exhibits reduced carboxylase and enhanced oxygenase function, resulting in an approximately 50% loss of carbon from metabolic pathways [2]. As a result, the efficiency of C3 photosynthetic activity decreases, and other metabolic processes such as photorespiration are enhanced [3]. Inorganic carbon not only participates in Calvin–Benson–Bassham (CBB) (“dark metabolism”) reactions, but also interacts with the participants of the “light stage” through the effect of HCO_3_^−^ (or CO_2_) on the transfer of electrons on both the donor and acceptor sides of photosystem II (PS II), the so-called “bicarbonate effect” [4,5,6]. Carbon dioxide flows in living cells are quite intense.A delay in inorganic carbon supply can not only slow down the process of photosynthesis, but can also seriously alter cell homeostasis and even cause cell death. Therefore, some plants require inorganic carbon concentration mechanisms in cells in proximity to RuBisCO when adapting to growth conditions during photosynthesis. These metabolic pathways, which vary among different groups of organisms, are called CO_2_-concentrating mechanisms (CCMs) (for a review, see [7]). Although the concentration of CO_2_ in aquatic environments is approximately the same as in air, its diffusion rate in water is 1000 times slower [8,9]. Thereby, aquatic photoautotrophs (such as cyanobacteria and algae) without CCMs can often be deficient in CO_2_ for photosynthesis [10]. In terrestrial higher plants, CCMs exist in the form of C4 photosynthesis with the primary carboxylation reactions and the Calvin cycle separated in space or time, as in the case of CAM metabolism.

Research on photosynthesis remains important in the modern world, as it examines issues such as the increase in photosynthesis productivity among agricultural plants through enhancing the efficiency of RuBisCO and dark metabolism reactions, as well as reducing the loss of inorganic carbon during fixation. Studies of photosynthetic electron transport chain functioning and the enzymes involved in dark and light reactions in photosynthesis remain relevant. The investigation of photosynthetic processes in tree and shrub plant species enables the development of effective strategies for increasing the sustainability and biodiversity of forests in adverse environmental conditions worldwide.

## 2. The Topics Considered in This Special Issue

Several studies published in the Special Issue involve research into the photosynthetic characteristics of various species that form forest ecosystems. The researchers aimed to promote the ecological stability of various trees and shrubs in adverse environmental conditions, as well as to develop strategies for improving the productivity of forest species.

### 2.1. Photosynthesis, Anatomy, and Metabolism as a Tool for Assessing Physiological Modulation in Five Native Species of the Brazilian Atlantic Forest [11]

The Brazilian Atlantic Forest is known as an exceptional biodiversity hotspot. The aim of the study was to examine the physiological and biochemical parameters of several native species of this forest to improve the conditions for their restoration and reveal the ecophysiological adaptations of forest habitats. The study found that some studied species, such as *Clusia nemorosa* and *Paubrasilia echinata*, exhibit robust mechanisms to mitigate the adverse effects of drought, while others, such as *Schinus terebinthifolius* and *Chorisia glaziovii*, show good adaptability. This study provides a comprehensive overview of the strategies used by different forest species to mitigate water stress during the dry season.

### 2.2. Photosynthetic Induction Characteristics in Saplings of Four Sun-Demanding Trees and Shrubs [12]

The photosynthetic induction response under constant and fluctuating light conditions was investigated in seedlings of four sun-demanding woody species: *Eucalyptus* spp., *Ficus macrocarpa* L., *Hibiscus syriacus* L., and *F. carica* L., to determine the relationship between gas exchange parameters among the species during photosynthetic induction. The significant differences in the induction status and time of gas exchange parameters among the studied species, especially between the two shrubs *F. carica* and *H. syriacus* were found. The results of the study showed that, to enhance the carbon uptake increment of woody species under naturally dynamic light conditions, attention should be focused on improving the stomatal opening rate or initial stomatal conductance.

### 2.3. Improving Tree Seedling Quality Using Humates Combined with Bacteria to Address Decarbonization Challenges Through Forest Restoration [13]

The aim of the study was to improve the quality of reforestation for atmospheric carbon sequestration using humic substances in combination with rhizosphere microorganisms to stimulate the growth of seedlings of various woody plants. The authors assessed various morphological and photosynthetic parameters of plants and found that the effect depended on both the plant and bacterial species, identifying the most effective strains and the most susceptible forest-forming species.

A number of published works are devoted to the characteristics of photosynthetic parameters of herbaceous plant species of agricultural importance.

### 2.4. Comparative Insights into Photosynthetic, Biochemical, and Ultrastructural Mechanisms in Hibiscus and Pelargonium Plants [14]

A comparison of the physiological and metabolic strategies that optimize the photosynthetic efficiency of *Hibiscus rosa-sinensis* and *Pelargonium zonale* plants was performed. The authors found that Hibiscus exhibits excellent photosynthetic productivity, which is supported by its robust chloroplast architecture and leaf anatomical features that allow the efficient capture and utilization of light energy during photosynthesis. At the same time, Pelargonium plants, which have a high mitochondrial content and less efficient chloroplasts, favor alternative metabolic pathways. The authors emphasize that the results of the study have implications for agriculture when selecting cultivated species depending on potential stressful growing conditions.

### 2.5. Pathways of Oxygen-Dependent Oxidation of the Plastoquinone Pool in the Dark After Illumination [15]

The authors analyzed the biphasic characteristics of plastoquinone (PQ) pool oxidation in isolated pea thylakoids by measuring OJIP kinetics. A “fast” phase is presumably related to the flow of electrons from the PQ pool to downstream acceptors of the photosynthetic electron transport chain, whereas a “slow” phase occurs due to the oxidation of PQH_2_ through oxygen-dependent mechanisms. A thorough analysis of the “slow” phase revealed the contribution of plastid terminal oxidase (PTOX) and H_2_O_2_ in modulating the redox state of the PQ pool in plant thylakoids under different light conditions, indicating their important role in regulating chloroplast metabolism.

### 2.6. Non-Foliar Photosynthesis in Pea (Pisum sativum L.) Plants: Beyond the Leaves to Inside the Seeds [16]

This study examines photosynthesis in non-foliar tissues—the pericarp, sheath, and cotyledon organs of *Pisum sativum* L. plants at the middle stage of seed maturation—to provide insights into the mechanisms by which non-foliar green plant tissues adapt to efficiently capture and utilize light under low-light conditions. The authors concluded that non-foliar tissues are capable of photosynthesis even under low-light conditions, which likely allows plants to produce additional energy.

The enzyme carbonic anhydrase (CA) is of special importance for the metabolism of autotrophs. These enzymes accelerate the interconversion of CO_2_ and bicarbonate with the release and consumption of a proton, enhancing the incorporation of inorganic carbon into organic compounds. Several works published in the Special Issue are devoted to studies of these enzymes.

### 2.7. The Freshwater Cyanobacterium Synechococcus elongatus PCC 7942 Does Not Require an Active External Carbonic Anhydrase [17]

A transformant of *Synechococcus elongatus* with expressed homologous of periplasmic carbonic anhydrase EcaACya from *Cyanothece* sp. ATCC 51142 was created. Most of the experiments revealed no substantial differences between wild-type and transformant cells. The authors concluded that the active external CA in the transformant cells contributed to a more rapid removal of CO_2_ from the medium. This resulted in the earlier onset of inorganic carbon-limiting conditions in transformants than in the wild-type. The main conclusion of the research is that *Synechococcus* normally does not require the presence of active external CA.

### 2.8. Highly Active Carbonic Anhydrase of the Thylakoid Lumen of Chlamydomonas reinhardtii [18]

CAH3 is a CA of the green alga *C. reinhardtii*, located in the thylakoid lumen [19]. This CA may participate in the CCM mechanism by supplying CO_2_ for RuBisCO in the pyrenoid [10], or by accelerating the removal of protons from the active center of the water-oxidizing complex of PSII, thus maintaining maximum photosynthetic activity [20]. In the study [18], the researchers investigated the properties of the recombinant CAH3 protein. This CA showed more than 3-fold higher activity compared to the other CA from the same organism, CAH1, and more than 11-fold higher activity compared to previous studies with recombinant CAH3. The characteristics of recombinant CAH3 were studied, including thermal stability at different pH values, sensitivity to inhibitors, and cations. The study is also the first to characterize the esterase activity of this enzyme.

### 2.9. Effect of the Absence of α Carbonic Anhydrase 2 on the PSII Light-Harvesting Complex Size in Arabidopsis thaliana [21]

This work continues the characterization of the effects of the knockout of the α-CA2 encoding gene in *Arabidopsis thaliana* plants that was previously started by the same research group [22]. The work published in the present Special Issue shows that the absence of α-CA2 in *A. thaliana* knockout mutants led to an increase in the size of the photosystem II light-harvesting antenna (LHCII) [21]. It is assumed that this effect was due to the decrease in the content of hydrogen peroxide in the leaves of plants with *α-CA2* gene knockout, affecting LHCII size through chloroplast retrograde H_2_O_2_ signaling pathways. The authors concluded that the changes in Arabidopsis plants induced by α-CA2 knockout indicate that this CA is localized in the chloroplasts of these plants.

The reviews published in the Special Issue cover various aspects of photosynthetic metabolism, including modern approaches to improving crop productivity.

### 2.10. Inorganic Carbon Acquisition and Photosynthetic Metabolism in Marine Photoautotrophs: A Summary [23]

The review is devoted to the continuing topicality of the problem of inorganic carbon concentration in marine inhabitants, cyanobacteria, algae, and sea grasses. It summarizes the current understanding of photosynthetic carbon assimilation by submerged marine photoautotrophs and, in particular, how their “biophysical” mechanisms of CO_2_ concentration differ from the “biochemical” types that take pace in terrestrial plants that use C4 photosynthesis and plants with Crassulacean Acid Metabolism.

### 2.11. Assembly and Repair of Photosystem II in Chlamydomonas reinhardtii [24]

The photosystem II assembly in *Chlamydomonas reinhardtii* provides an excellent model system for the evolution of and interplay between nuclear and organellar genomes. The mechanisms by which PSII is assembled and turned over in the green alga *C. reinhardtii* are reviewed. Important questions about the assembly and repair of active PSII dimers in the cells of this widely studied model organism are discussed. The authors consider the evolution of photosynthetic organisms from the perspective of the pathways involved in the phosphorylation, dephosphorylation, and degradation of PSII in these algae, as these pathways demonstrate intermediate mechanisms between cyanobacteria and plants.

### 2.12. Improving Crop Yield Through Increasing Carbon Gain and Reducing Carbon Loss [25]

The review summarizes strategies for increasing crop yields by enhancing photosynthetic efficiency in C3 plants, which were developed and tested for both model and agricultural crops, primarily due to the optimization of the RuBisCO enzyme. Prospects for introducing inorganic carbon concentrating mechanisms and manipulations using photorespiration bypasses into higher C3 plants are also considered. The authors note that despite existing advances, the relationship between abiotic stresses and photosynthesis is poorly understood and needs to be clarified, which is critical to fully exploiting the potential improvements achieved through these methodologies.

## 3. Conclusions

The Special Issue “Photosynthesis and Carbon Metabolism in Higher Plants and Algae” collates research papers on all aspects of photosynthesis in terrestrial organisms and aquatic habitants: in higher plants, algae, and procaryotic photoautotrophs–cyanobacteria. The papers are devoted to the specificity of inorganic carbon transport into their cells and organoids, carbon metabolism, and electron transport chain functioning.

The results published in the Special Issue shed light on the functioning of leaf and non-leaf photosynthesis in the tissues of woody and herbaceous species of higher plants, and in the cells of algae and cyanobacteria. The locations, functions, isolation, properties, and structure of photosynthetic enzymes, as well as of the organization of the photosynthetic light-harvesting antenna and cytochrome *b*_6_*f* complex, are also explored. Advances in the understanding of inorganic carbon concentration mechanisms in lower plants, strategies for increasing CO_2_ consumption productivity in higher C3 plants, and increasing the environmental sustainability and biodiversity of forest ecosystems are also reviewed.

## References

[1] Parry M.A.J., Andralojc P.J., Mitchell R.A.C., Madgwick P.J., Keys A.J. (2003). Manipulation of Rubisco: The amount, activity, function and regulation. J. Exp. Bot..

[2] Kannappan B., Gready J.E. (2008). Redefinition of Rubisco carboxylase reaction reveals origin of water for hydration and new roles for activesite residues. J. Am. Chem. Soc..

[3] Sage R.F. (2002). Variation in the k(cat) of Rubisco in C3 and C4 plants and some implications for photosynthetic performance at high and low temperature. J. Exp. Bot..

[4] Allakhverdiev S.I., Yruela I., Picorel R., Klimov W. (1997). Bicarbonate is an essential constituent of the water-oxidizing complex of photosystem II. PNAS.

[5] Klimov V.V., Baranov S.V. (2001). Bicarbonate requirement for the water-oxidizing complex of photosystem II. BBA.

[6] Van Rensen J.J., Tonk W.J.M., Bruijn S.M. (1988). Involvement of bicarbonate in the protonation of the secondary quinone electron acceptor of Photosystem II via the non-haem iron of the quinone-iron acceptor complex. FEBS Lett..

[7] Raven J.A., Beardall J., Sánchez-Baracaldo P. (2017). The possible evolution and future of CO_2_-concentrating mechanisms. J. Exp. Bot..

[8] Walker N.A. (1983). The uptake of inorganic carbon by freshwater plants. Plant Cell Environ..

[9] van Iperen J., Helder W. (1985). A method for the determination of organic carbon in calcareous marine sediments. Mar. Geol..

[10] Moroney J.V., Ynalvez R.A. (2007). Proposed carbon dioxide concentrating mechanism in *Chlamydomonas reinhardtii*. Eukaryot. Cell.

[11] Rodríguez-Páez L.A., Seleiman M.F., Alhammad B.A., Pineda-Rodríguez Y.Y., Pompelli M.F., Martins A.O., Dias-Pereira J., Araújo W.L. (2024). Photosynthesis, Anatomy, and Metabolism as a Tool for Assessing Physiological Modulation in Five Native Species of the Brazilian Atlantic Forest. Plants.

[12] Liu Q., Jin W., Huang L., Wang D., Xu K., Wei Y. (2025). Photosynthetic Induction Characteristics in Saplings of Four Sun-Demanding Trees and Shrubs. Plants.

[13] Nazarov A., Chetverikov S., Timergalin M., Ivanov R., Ryazanova N., Shigapov Z., Tuktarova I., Urazgildin R., Kudoyarova G. (2024). Improving Tree Seedling Quality Using Humates Combined with Bacteria to Address Decarbonization Challenges through Forest Restoration. Plants.

[14] Falcioni R., Antunes W.C., de Oliveira R.B., Chicati M.L., Demattê J.A.M., Nanni M.R. (2024). Comparative Insights into Photosynthetic, Biochemical, and Ultrastructural Mechanisms in Hibiscus and Pelargonium Plants. Plants.

[15] Naydov I., Kozuleva M., Ivanov B., Borisova-Mubarakshina M., Vilyanen D. (2024). Pathways of Oxygen-Dependent Oxidation of the Plastoquinone Pool in the Dark After Illumination. Plants.

[16] Stepanova N., Zhilkina T., Kamionskaya A., Smolikova G. (2024). Non-Foliar Photosynthesis in Pea (*Pisum sativum* L.) Plants: Beyond the Leaves to Inside the Seeds. Plants.

[17] Kupriyanova E.V., Sinetova M.A., Gabrielyan D.A., Los D.A. (2024). The Freshwater Cyanobacterium *Synechococcus elongatus* PCC 7942 Does Not Require an Active External Carbonic Anhydrase. Plants.

[18] Terentyev V.V., Trubitsina L.I., Shukshina A.K., Trubitsin I.V., Rudenko N.N. (2025). Highly Active Carbonic Anhydrase of the Thylakoid Lumen of *Chlamydomonas reinhardtii*. Plants.

[19] Karlsson J., Ciarke A.K., Chen Z.Y., Hugghins S.Y., Park Y.I., Husic H.D., Moroney J.V., Samuelsson G. (1998). A Novel α-Type Carbonic Anhydrase Associated with the Thylakoid Membrane in *Chlamydomonas reinhardtii* Is Required for Growth at Ambient CO_2_. EMBO J..

[20] Terentyev V.V., Shukshina A.K. (2024). CAH3 from *Chlamydomonas reinhardtii*: Unique Carbonic Anhydrase of the Thylakoid Lumen. Cells.

[21] Nadeeva E.M., Rudenko N.N., Ignatova L.K., Vetoshkina D.V., Ivanov B.N. (2025). Effect of the Absence of α Carbonic Anhydrase 2 on the PSII Light-Harvesting Complex Size in *Arabidopsis thaliana*. Plants.

[22] Nadeeva E.M., Ignatova L.K., Rudenko N.N., Vetoshkina D.V., Naydov I.A., Kozuleva M.A., Ivanov B.N. (2023). Features of Photosynthesis in *Arabidopsis thaliana* Plants with Knocked Out Gene of Alpha Carbonic Anhydrase 2. Plants.

[23] Beer S., Beardall J. (2025). Inorganic Carbon Acquisition and Photosynthetic Metabolism in Marine Photoautotrophs: A Summary. Plants.

[24] Mehra H.S., Wang X., Russell B.P., Kulkarni N., Ferrari N., Larson B., Vinyard D.J. (2024). Assembly and Repair of Photosystem II in *Chlamydomonas reinhardtii*. Plants.

[25] Karthick P.V., Senthil A., Djanaguiraman M., Anitha K., Kuttimani R., Boominathan P., Karthikeyan R., Raveendran M. (2024). Improving Crop Yield through Increasing Carbon Gain and Reducing Carbon Loss. Plants.

